# Initial characterization of the human central proteome

**DOI:** 10.1186/1752-0509-5-17

**Published:** 2011-01-26

**Authors:** Thomas R Burkard, Melanie Planyavsky, Ines Kaupe, Florian P Breitwieser, Tilmann Bürckstümmer, Keiryn L Bennett, Giulio Superti-Furga, Jacques Colinge

**Affiliations:** 1 CeMM - Center for Molecular Medicine of the Austrian Academy of Sciences, Lazarettgasse 19/3, A-1090 Vienna, Austria

## Abstract

**Background:**

On the basis of large proteomics datasets measured from seven human cell lines we consider their intersection as an approximation of the human central proteome, which is the set of proteins ubiquitously expressed in all human cells. Composition and properties of the central proteome are investigated through bioinformatics analyses.

**Results:**

We experimentally identify a central proteome comprising 1,124 proteins that are ubiquitously and abundantly expressed in human cells using state of the art mass spectrometry and protein identification bioinformatics. The main represented functions are proteostasis, primary metabolism and proliferation. We further characterize the central proteome considering gene structures, conservation, interaction networks, pathways, drug targets, and coordination of biological processes. Among other new findings, we show that the central proteome is encoded by exon-rich genes, indicating an increased regulatory flexibility through alternative splicing to adapt to multiple environments, and that the protein interaction network linking the central proteome is very efficient for synchronizing translation with other biological processes. Surprisingly, at least 10% of the central proteome has no or very limited functional annotation.

**Conclusions:**

Our data and analysis provide a new and deeper description of the human central proteome compared to previous results thereby extending and complementing our knowledge of commonly expressed human proteins. All the data are made publicly available to help other researchers who, for instance, need to compare or link focused datasets to a common background.

## Background

The understanding of living cells at a systemic level is being recognized more and more as an important component of biology and medicine research [1-9]. Biological pathways and networks of protein interactions are key paradigms to link molecules to biological functions and by so doing bridging the genotype-to-phenotype gap as well as understanding properties of the organization of biological matter [10-13]. In this work we aim at answering three simple but fundamental questions: i) What is the complement of human proteins expressed ubiquitously and abundantly in different cell types? ii) Does this *central proteome *(C.Prot) [14] display properties that are distinct from the rest? iii) Can one identify global features of this central proteome?

Gene expression microarrays allow analyzing a large variety of transcriptomes [15] and several studies using mRNA detection or abundance as a proxy for protein expression or concentration have revealed important properties of gene sets related to tissue specificity [16-18]. Recently, Bossi and Lehner [19] showed that tissue-specific proteins are less interacting but bind to core cellular components and common proteins. Domains enriched in tissue-specific genes tend to be metazoan-specific and are non-essential [20]. It is also known that widely expressed genes encode protein domains involved in protein degradation, cytoskeleton or RNA-binding [20].

It is well known that correlation between transcripts and protein abundance is variable [21] and, as a general rule of thumb, a good correlation is observed in one third of the observed entities only. Subsequent mechanisms of regulation can significantly decouple protein and transcript abundance [22]. For this reason, we believe that it is important to study the central proteome from proteomics data directly. As our data show, mass spectrometry sensitivity has achieved a level that permits such direct approaches. Similar work was conducted by Schirle, et al. [14], who first coined the term central proteome and used human cell lines as we did, though they limited their analysis to technical aspects related to the proteomics technology. Kislinger, et al. [23] profiled protein expression in six mouse organs. Another related project is the Human Protein Atlas [24] that maps protein expression in human tissues through a selected set of antibodies.

The focus of our work is different compared to the aforementioned transcriptomics and proteomics studies. After a brief and classical analysis of the functions of the proteins present in the central proteome, which matches gene microarray results, we reveal important new findings regarding the gene structures of genes coding the central proteome, location on pathways in relation with drug targets, and global properties of the interaction network connecting the central proteome. Furthermore, we show how several characteristics of common proteins vary with protein abundance.

The large amount of data generated for this research constitutes a unique and homogeneous dataset that should interest other investigators. Data are made available as supplementary material and are accessible from the ProteomeCommons.org Tranche public repository.

## Results

### Cell lines, proteomics and protein identifications

We measured the proteomes of seven cell lines from the three germ layers (HaCat, HepG2, K562, HEK293, Namalwa, U937, HeLa) with 1D SDS-Page followed by LC-MS/MS. The proteomes contained between 2031 and 4154 proteins each (see Table 1). Protein identification was accomplished by a bioinformatics platform combining two database search engines, Mascot [25] and Phenyx [26], and an innovative and very stringent validation strategy enforcing a maximum false discovery rate (FDR) of 0.25% on protein groups [27]. In addition, protein groups that were not made of alternative splice variants exclusively (2%) were discarded. Specific peptides allowed us to ascertain the presence of some variants.

**Table 1:** Number of protein groups and distinct peptides identified in the proteomics data.

Cell line	Protein groups	**With isoforms**^**a**^	**Specific isoforms**^**b**^	Distinct peptides	Germ layer
HaCat	2031	2673	13	29040	Ectoderm
HEK293	4154	5412	64	71571	Mesoderm
HeLa	2379	3075	31	31609	Mesoderm
HepG2	2494	3298	24	30194	Endoderm
K562	3141	4078	37	48202	Mesoderm
Namalwa	2686	3527	29	37512	Mesoderm
U937	2073	2720	25	28786	Mesoderm

(a) Number of protein group reporters in case alternative splice variants are counted as well because they have all the peptides of the protein group, although they do not necessarily have a specific peptide detected. (b) Isoforms supported by a specific peptide.

Each cell line was analyzed twice in technical replicates (merged results in Table 1) and modest variability in the identified proteins was observed (<4%).

### The central proteome

A large number of proteins were identified in each cell line (Table 1). We constructed the *central proteome *(C.Prot) by selecting proteins found in all the 7 cell lines, i.e. 1124 proteins. HEK293 cell line yielded notably more protein identifications, which was observed by others already [14]. In addition, as mentioned above, technical replicates of all the cell lines were highly reproducible. HEK293 higher number of proteins is hence unlikely to have been caused by experimental bias. In fact, HEK293 cells provide a convenient system for expressing many proteins, notably in affinity purification MS experiments [28].

We compared the proteins identified in the 7 cell lines and C.Prot with all the human proteins listed in UniProtKB/Swiss-Prot [29] to determine possible experimental biases in molecular weight, isoelectric point, hydrophobicity, and aliphatic index [30]; see Additional file 1: Suppl. Figure 1. We observed very modest shifts towards larger (+2.3% on average) and more acidic proteins (-5.5%) in C.Prot. These slight biases are consequences of the analytical technologies used and we do not believe they have any significant impact on what follows.

Overlap between C.Prot and the Human Protein Altlas (HPA) was of less than 40%, depending on HPA detection strength. Namely, HPA contains all our cell lines but Namalwa and we considered the intersection of the 6 shared cell lines in HPA. We found 852 proteins strongly expressed (178 of which are in C.Prot, 16% of C.Prot) and 3314 at least weakly expressed (413 in C.Prot, 37%). We also considered all (union) the proteins expressed in all the 46 HPA cell lines, weakly or better, and we found 4314 proteins (445 in C.Prot, 40%).

Accession codes, IDs, description, and gene names of the 1124 proteins found in the central proteome are provided as Additional file 2.

### Main functions represented in the central proteome

We classified the main categories of proteins present in C.Prot by using a slimmed gene ontology (generic GO slim) [31] and KEGG [32,33]. GO term statistics were obtained via topGO [34] with a 5% cutoff on the P-values ("weight.log" method).

C.Prot was mainly enriched for vital processes of the cell, see Table 2 and Suppl. Figure 2. The GO categories broadly ranged from proteostasis, such as translation and protein transport, over metabolic processes to cell cycle and death. The metabolic processes mainly consisted of primary metabolism, which is vital for maintenance and proliferation. Major catabolic KEGG pathways covered by C.Prot were proteasome, citrate cycle, oxidative phosphorylation, glycolysis/gluconeogenesis, phosphate pentose pathway, fatty acid degradation and few amino acid degradation pathways. On the biosynthetic side we found translation, most aminoacyl-tRNA biosynthesis enzymes and pyrimidine metabolism. The GO category "cellular component organization and biogenesis" contained important complexes such as signal recognition particle, coatomer protein complex and the splicosome. Finally, the cell cycle together with DNA metabolism and cell death, which are vital functions of living in a cellular population, were also enriched. The same broad coverage could be observed for the molecular functions and the cellular localizations in C.Prot, except for the plasma membrane and the extracellular space due to proteomics technical limitations and use of cell lines, see Suppl. Figures 3 and 4.

**Table 2:** Gene ontology terms (biological process) found significant at the 5% level in the central proteome.

GO ID	GO Term	P-value	**Coverage**^**a**^
GO:0006412	translation	1.00E-30	0.37
GO:0016043	cellular component organization and biogenesis	1.40E-29	0.13
GO:0015031	protein transport	1.90E-25	0.19
GO:0043170	macromolecule metabolic process	5.10E-23	0.14
GO:0009056	catabolic process	3.60E-19	0.15
GO:0044238	primary metabolic process	1.00E-16	0.10
GO:0006091	generation of precursor metabolites and energy	2.90E-14	0.25
GO:0007049	cell cycle	5.10E-12	0.14
GO:0006259	DNA metabolic process	4.30E-07	0.13
GO:0009058	biosynthetic process	7.50E-06	0.20
GO:0009719	response to endogenous stimulus	6.10E-04	0.12
GO:0008219	cell death	8.90E-04	0.10
GO:0019725	cellular homeostasis	2.95E-03	0.12
GO:0006950	response to stress	4.30E-03	0.10

^a^Coverage indicates the proportion of proteins annotated in Swiss-Prot with the term that are found in C.Prot.

It is worth noting that advanced GO analysis supported by the topGO R package, where detailed GO annotations are reported to ancestor terms if they do not yield significant results only, combined with generic GO slim helped considerably dealing with a large dataset such as C.Prot. Classical enrichment analysis methods overvalued very general categories and/or returned numerous very detailed hits. We found the "weight.ratio" method of topGO too stringent and the "classic" one too "verbose".

### A well conserved set of proteins coded by exon rich genes

The presence of a protein in many species is an indicator of high conservation and fundamental functional role. Therefore, we queried all orthologs of the human genome from Ensembl and we measured conservation by counting, for each human protein, the number of species that had one ortholog. We found that, on average, C.Prot proteins had 5.9 orthologs more than human proteins taken from SwissProt (P = 0, Wilcoxon), that is they were present in more species thereby indicating their higher degree of conservation.

To contrast our results we wanted to compare with cell specific proteins. Due to the limited number of cell lines available it was not feasible to define such specific proteins. We hence used tissue specific genes as a proxy [15]. In the Su et al. dataset, testis genes contributed for almost one third and hence we defined a specific transcriptome (Spe.Trans) ignoring testis specific. As a matter of fact, such diversity in testis gene expression would hide gene specificity in many other tissues otherwise. Without testis genes, Spe.Trans contained 282 genes that, on average, had -0.6 orthologs (P = 0.06) compared to the reference Swiss-Prot database.

An important feature of eukaryote proteins is that they have the possibility to exist in different splice variants. The total count of exons indicates combination diversity. Remarkably, in C.Prot, the exon count was significantly shifted towards higher values with an average shift as large as +3.7 (P = 0, Wilcoxon). Conversely, Spe.Trans had -0.7 exons on average (P = 0.5). To prevent a potential bias in exon counts through a slight bias in protein sequence lengths in our data, we compared exon counts in C.Prot with Swiss-Prot applying a bootstrap procedure to draw appropriate sequence length distributions from Swiss-Prot.

### A well connected and central central proteome

**Figure 1:**
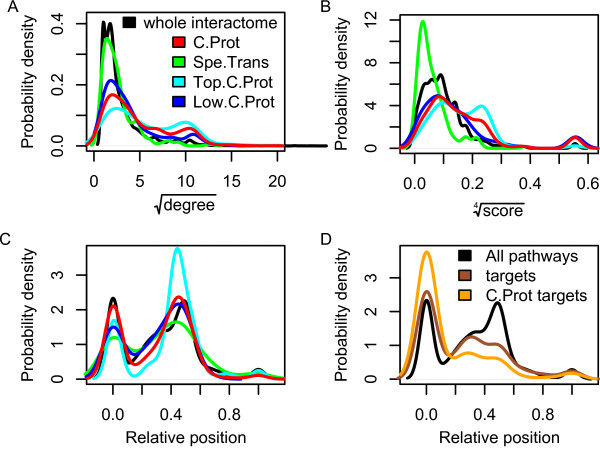
**Network and pathways statistics**. (A) Node degree (number of edges). Note the strong shift of C.Prot towards higher values. We also observe the absence of shift of the tissue specific genes (Spe.Trans) and the gradual shift from low abundant C.Prot entities to high abundant ones. (B) Eigenvector centrality values also display similar shifts, although in this case Spe.Trans even reverses the trend and differences between low and high abundant C.Prot are more modest. (C) Relative positions in pathways; 0 = beginning, 1 = end. No real bias for C.Prot but a strong preference for central position for its abundant proteins. Spe.Trans and low abundant C.Prot are more spread over all possible positions. (D) The same for drug targets. Note the strong shift towards initial positions for C.Prot drug targets, which significantly amplifies the already present preference of drug targets for such positions.

Using an integrated interaction database, we extracted a human interactome comprising 9,495 proteins and 70,083 interactions. We computed 5 commonly used connectivity measures (betweenness and eigenvector centrality, clustering coefficient, degree, k-core score) to reflect various local and global aspects of the interactome topology at the positions occupied by C.Prot. All 5 measures indicated significant positive biases, i.e. more centrality, higher connectivity, and more frequent participation in protein complexes. Remarkably, a further significant increase for abundant C.Prot proteins was observed. Node degree and eigenvector centrality statistics appear in Figures 1A and 1B as examples.

There was a significant association of C.Prot with drug targets as listed in DrugBank [35] (176 targets among the 1465 listed, P = 1.6E-23, bootstrapped χ^2 ^see Methods). In the human interactome, we observed that drug targets were more central and more connected nodes as reported previously by others [8]. We did not see a consistent increase of this trend with targets restricted to C.Prot. On the contrary, considering relative positions in pathways (see Methods), drug targets in C.Prot displayed a significantly different profile compared to targets in general. Targets not restricted to C.Prot appeared to be preferentially at the beginning of pathways as opposed to more central positions (P < 3.0E-2). When restricted to C.Prot, this trend was much augmented (P < 1.4E-34). NCI-PID [36], the pathway database we used, included 759 DrugBank targets (21%), 133 of which were in C.Prot (12% of C.Prot).

### A central interactome

**Figure 2:**
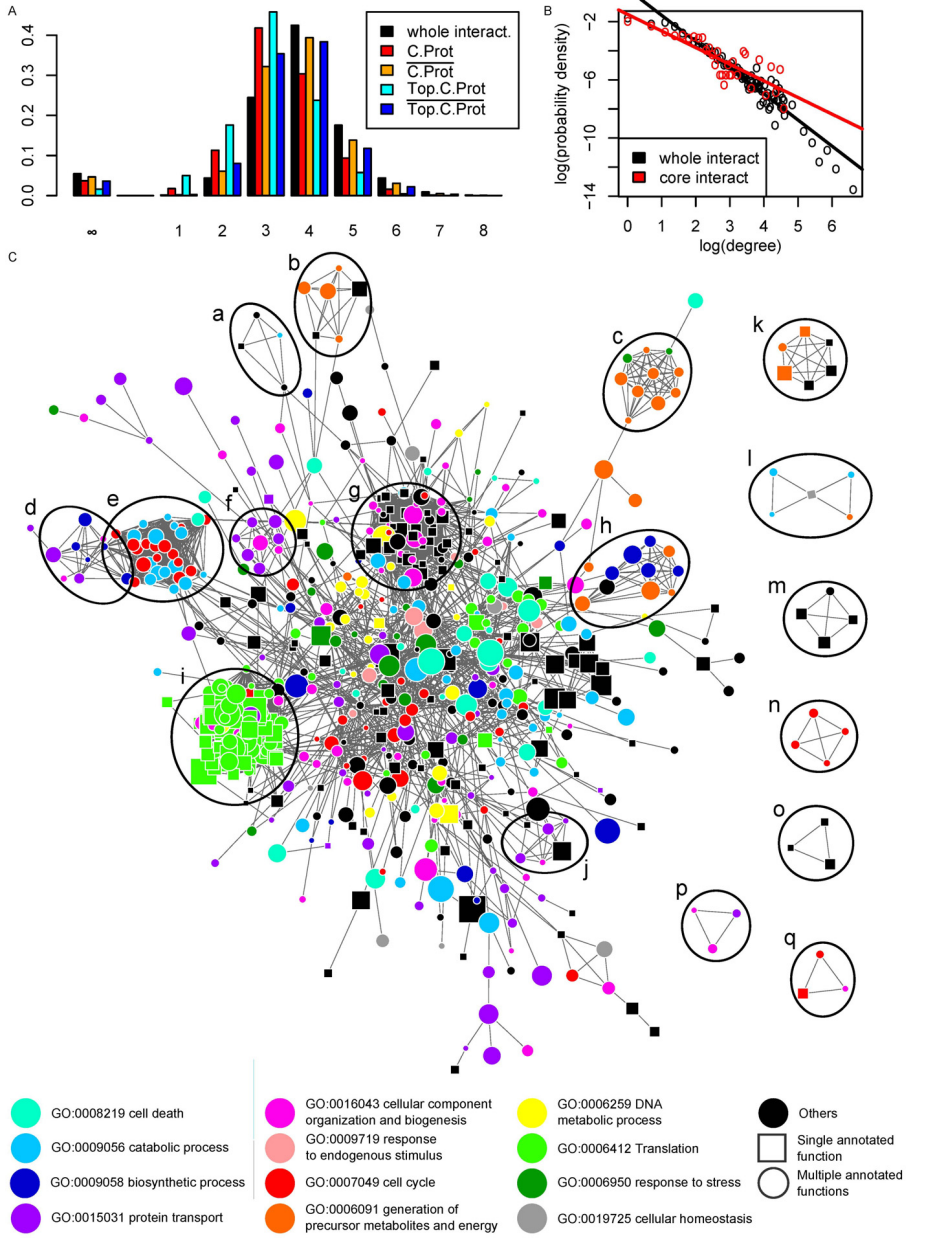
**The central interactome**. (A) Shortest path distance distributions. We first remark that distances between C.Prot entities (red) are closer than distances between proteins of the human interactome (black), i.e. short distances below 4, which is the mean and median distance, are over-represented. Remarkably, C.Prot is also closer than on average to the non C.Prot proteins (orange). The abundant C.Prot proteins are even closer to each other and to the non C.Prot proteins (cyan and blue). It shows that C.Prot (and its most abundant components) are embedded "uniformly" in the human proteome. (B) Power law distribution of the whole human interactome versus the central interactome. The central interactome is more connected (exponent -1.1), i.e. frequency of high node degrees decreases slower, than the whole (exponent -1.8). (C) Central interactome with mapped significant biological processes (Table 2). Processes not significantly enriched in C.Prot are in black and multiple GO annotations are depicted by a circle (color chosen randomly) as opposed to a square for single GO. Shared GO term ancestors at a node were removed to eliminate trivial multiple annotations and stay at the most specific levels. We note that, except for a few, processes are not strongly localized in this network. It does not represent juxtaposed pathways but rather an exchange platform. We also observe that most proteins have multiple GO BP annotations (circular node shape), which *de facto *establish additional exchanges between fundamental cellular processes. Finally, we recognized some important complexes: (a) exosome, (b) ubiquitinol-cytochrome c reducatase, (c) NADH dehydrogenase, (d) oligosaccharyl transferase, (e) proteasome, (f) COPI, (g) ribonucleoprotein/splicosome, (h) proton-transporting ATP synthase, (i) ribosome, (j) signal recognition particle, (k) cytochrome c oxidase subunits, (l) pyruvate/2-oxoglutarate dehydrogenase complex, (m) prefoldin, (n) condensin, (o) Signal peptidase complex, (p) COPII, (q) septin complex. Network visualized with Cytoscape [56].

**Figure 3:**
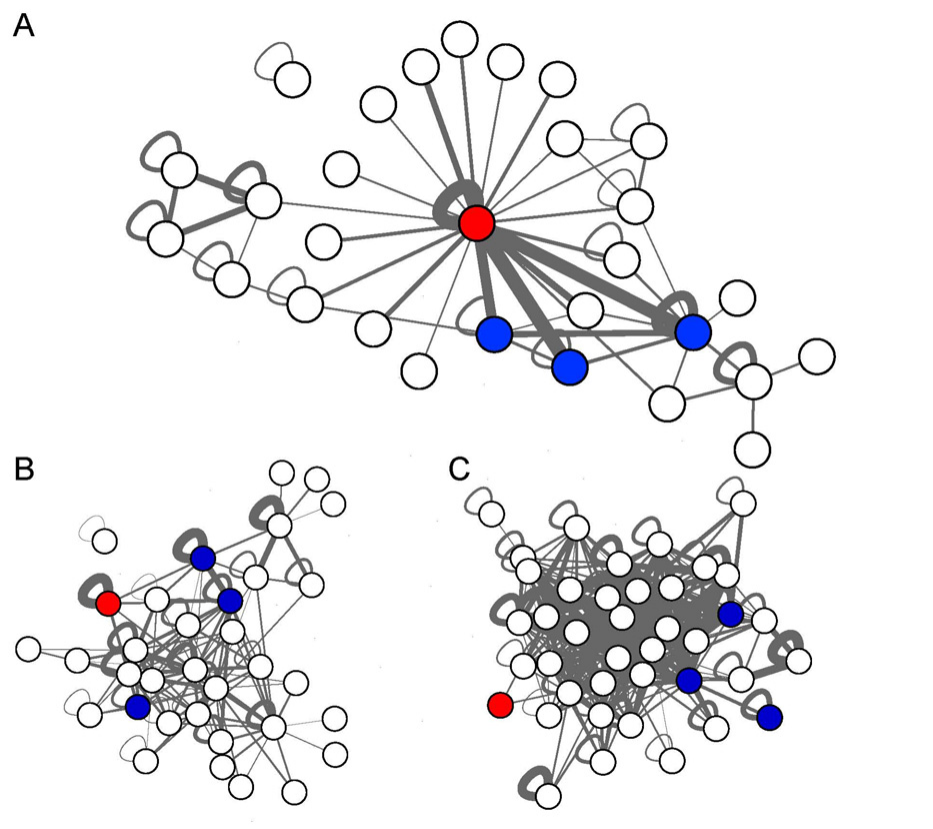
**Inter-biological process exchanges over the central interactome**. High-scoring fluxes between biological processes provide us with a mean to summarize the main function of the central interactome, a subset of the human interactome that is likely to be expressed in all the human cells. In our scoring scheme, high scores represent fluxes that are much more intense than expected from GO term frequencies and protein connectivity, i.e. exchanges significantly favored by protein interactions. GO biological processes are represented as nodes and scores by the edge thickness. (A) Fluxes within the central interactome. The star-like topology with translation (red) at its center shows that most exchanges synchronize other cellular processes with translation. The strongest crosstalk can be observed between translation and GO categories (blue), which contain many members of the nucleic acid metabolism (needed for mRNA generation) and complexes such as signal recognition particle, coatomer protein complex and the splicosome. (B) Fluxes between C.Prot proteins and proteins not in C.Prot. As soon as the focus shifts away from the central interactome, translation loses its role as central communicator. Communication between C.Prot and non C.Prot are less specialized. Also, note the lost interconnectivity of the blue cluster, which reflects reduced activity of the processes mentioned above. (C) This trend is further amplified in the external fluxes between proteins not in C.Prot that become essentially global and ignore translation.

It was natural to define the *central interactome *as the network made of direct interactions between C.Prot proteins; this network is likely to exist in all the human cells. Shortest path distance distributions were computed (Figure 2A). We also found both the central and whole interactome to be scale-free [37], with the central interactome comprising more highly-connected regions (protein complexes), see Figure 2B. The central interactome contained several essential protein complexes, see Figure 2C. The central interactome can be regarded as platform used by biological processes to exchange information through protein interactions. Therefore, we introduced a notion of flux between biological processes. As explained in Methods, fluxes between biological processes (BPs) mediated by protein-protein interactions (PPIs) can be scored and we compared fluxes over the central interactome with fluxes outside the central interactome and fluxes between C.Prot and non C.Prot proteins. In each case, we generated random interaction networks and GO annotations to determine which fluxes were significantly more intense than what would be expected by chance from the network topology and GO terms frequencies. At the 1% significance level, the random networks simulation yielded 57 significant GO BP fluxes within the central interactome, 135 between C.Prot and proteins of the human interactome not in more than 5 cell lines, and 365 outside the central interactome. In every case, the expected number was 12.75. See Figure 3.

## Discussion

### The central proteome dataset

We present results characterizing the human central proteome (C.Prot), i.e. the set of proteins commonly expressed by human cells. Although previous related studies have been conducted successfully on the basis of transcriptomics data, we based our work on proteomics data. Proteomics is likely to yield additional insight because it directly measures the entities of interest, provided it reaches sufficient sensitivity and does not introduce excessive experimental biases. Moreover, our results comprise several important new findings never covered by transcriptomics studies.

We defined C.Prot to be the proteins shared by 7 cell lines whose total cell lysates were analyzed by state of the art proteomics and followed by very stringent protein bioinformatic identifications; it contains 1124 proteins. Clearly, the intersection of a much larger number of cell lines would be too stringent a criterion but we found it appropriate with 7 cell lines. Experimental biases were modest in our data (Suppl. Figure 1).

We could not find comparable human datasets from public repositories [38,39] to complement our data and cover more cell lines or tissues. The dataset of Schirle, et al. [14] is much smaller after mapping to Swiss-Prot (5-10 times) and covered by our data essentially (>92%). The modest 8% that is not covered can certainly been explained by MS detection and sample preparation variability. Also, the few false positives contained in both datasets reduce the overlap further.

Data from the Human Protein Atlas [24] (HPA) are not appropriate for completing ours as they derive from a biased a priori selection of proteins and antibody availability. Antibody-based assays can also be very variable in their sensitivity, thus making expression profiles of different proteins difficult to compare. Nonetheless, HPA is a very valuable resource and comparison with our data showed that strong HPA common detections in 6 out of the 7 cell lines of our study available in HPA have similar sizes (852 proteins), whereas including weak detections increased this number dramatically to 3314. The overlap with C.Prot is modest in both cases: 178 and 413 proteins respectively (17% and 37%). This shows higher sensitivity detection of targeted antibody-based assays compared to broad unbiased MS analysis, which is no surprise. This also shows that HPA is not covering so far an important proportion of C.Prot.

HPA data can reveal an important characteristic of our data: if we consider all the proteins expressed in all the 46 HPA cell lines weakly or stronger, we find 4314 proteins (445 in C.Prot, 40%), which is a very small improvement compared to 413 above although HPA detections rose from 3314 to 4314. This indicates that, within the limitation of current MS detectability, we already identify with 7 cell lines only a significant part of the MS measurable central proteome. To improve coverage of the central proteome it is more important to improve MS sensitivity than to increase the number of cell lines.

We compared C.Prot with the most abundant proteins detected in any of the 7 cell lines that were not in C.Prot (see Methods). GO annotations were very different for the two datasets (Suppl. Figures 2-4). In fact, the non central abundant proteins had no strong functional association, which indicates that the sole abundance is not sufficient to associate with a function and is no longer a dominating factor for inclusion in the central proteome.

From Su et al. [15] microarray data we assembled a central transcriptome (C.Trans) for comparison taking the intersection of genes expressed in each tissue (commonly used MAS5 > 200), which resulted in a list of 2002 genes. C.Trans only covered 501 proteins of C.Prot. Su et al. chip contained 917 out of the 1124 proteins of C.Prot, meaning that 45% of the proteins we detected would be missed by a transcriptomics approach. Releasing the criterion for central transcriptome inclusion by requiring genes in all but 4 samples (instead of all), we increased its size to 3197 genes and cover 683 proteins, i.e. a missing part representing 26%. Such losses were most likely due to low degradation rate proteins that do not require permanent expression of their coding genes.

### Analysis

Functional analyses revealed prevalence for proteins involved in proteostasis, RNA-binding, primary metabolism, cell cycle and cell death (Table 2, Suppl. Figures 2 and 3). The transcriptomics data set of specific genes (Spe.Trans) [15] was associated with different biological processes related to signaling and multicellular organization, whereas vital biological processes were much less prevalent (Suppl. Figure 2). Spe.Trans thus seems to play an important role in the establishment of distinct anatomies via cell-cell signaling, whereas C.Prot covers the more "archaic" needs, what is supported by the higher degree of conservation of its members. These observations corroborate results found from transcriptomics data previously [20]. Proteins expressed across many cell types require augmented gene expression flexibility to adapt to local conditions. Our finding that C.Prot proteins have on average almost 4 additional exons compared to all human proteins shows that evolution preferred this economic way of gaining flexibility in common parts instead of augmenting their number through duplication events. Spe.Trans reduced exon count contrasts nicely.

Kislinger, et al. [23] did not extract a central proteome from their data. Taking the intersection of their 6 organ datasets and mapping the mouse proteins to their human orthologs, we obtained 393 Swiss-Prot entries, which are also biased towards high exon counts (+3).

Implication in central processes of the cell's life is naturally reflected by augmented PPIs and participation in complexes formation as well as central positions in interaction networks. Common proteins are more connected and central in the human interactome than on average as shown by the 5 different global and local network topology measures we computed. This trend is reversed for tissue specific genes and, in addition, we observed a significant dependence with protein abundance: the more abundant a protein the stronger the connectivity/centrality and *vice versa*, see Figures 1A and 1B. Such observations might be thought to be artifactual since common proteins are likely to be over-represented in interaction databases and abundantly expressed proteins might be more successful in PPI experiments. To exclude this potential confounding effect we mapped C.Prot down to yeast where large-scale unbiased data are available. Using our integrated database we obtained a yeast interactome comprising datasets published with at least 5000 physical interactions only and confirmed that C.Prot increased connectivity/centrality is still valid in yeast (P < 2.0E-7 for all 5 measures).

Because human interactome data have been gathered in many cell types and conditions, we do not believe that higher connectivity necessarily implies that C.Prot entities are permanently connected to more partners. We rather consider it as a strong indication that these proteins have gained, during evolution, the capability to bind to more partners when needed; another way to augment adaptability to various environments. This is corroborated by the top over-representation of the GO molecular function "protein binding", see Suppl. Figure 3.

To investigate robustness of our findings with respect to a less stringent definition of the central proteome, we checked the exon count and network statistics biases either using the intersection of any six cell lines or taking proteins found in 6 out of 7 cell lines. In every case, the results were almost unchanged.

After considering PPIs, it is natural to move to a higher degree of organization of the living matter, i.e. biological pathways. We define an intuitive notion of relative position along a pathway (0 = source, 1 = end point, see Methods and Suppl. Figure 5) and we observe that C.Prot positions just follow the typical distributions of human proteins (Figure 1C). This is no longer true for the most abundant common proteins, which tend to occupy central positions. This indicates that abundant proteins are more likely at non-rate limiting central positions while the key steps are under tighter expressional control [40]. This is also supported by more uniform presence of Spe.Trans genes and low abundant common proteins along pathways (Figure 1C).

**Figure 4:**
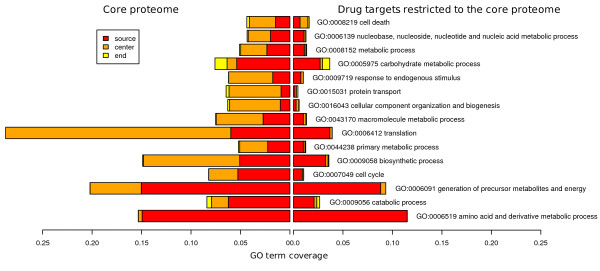
**Drug targets GO terms variation along pathways**. Integration of GO biological process (BP) analysis and pathway positions. Proteins at the source (0-0.2), center (0.4-0.6) and end (0.8-1) of pathways in C.Prot and drug targets restricted to C.Prot are submitted to GO analysis. All the BP terms with P-values < 0.1% in at least one case are reported and we see that the general strong reduction for central pathway position (Figure 1D) is rather uniform over the BPs. The barplots represent the coverage of the GO terms.

Where do drugs hit the central proteome? The size of the overlap between drug targets and C.Prot is significantly large (176 proteins, P = 1.6E-23). Generally, drug targets are mainly designed against catalytic proteins, transporters and receptors, see Suppl. Figures 3 and 4. Drug targets in C.Prot are mainly enzymes (146), which play a pivotal role in primary metabolic processes (130). The biological processes of amino acid metabolism, precursor generation and carbohydrate metabolism are strongly targeted. A preference against nucleotide binding proteins, e.g. 50 ATP binders, and electron carrier activity processes is observed. Pathway position analysis showed that drug targets are clearly shifted towards sources of pathways, see Figure 1D. This trend was strongly reinforced considering C.Prot drug targets. Figure 4 features GO analysis localized along pathway positions and shows that less central positions are targeted in all the biological processes. We believe that the reason for an additional shift towards initial pathway positions is that C.Prot targets contain metabolic proteins mostly. As a matter of fact, drugs targeting metabolic pathways are frequently designed against rate limiting enzymes, e.g. ATP- or NAD-binding proteins, which are found at the beginning of pathways.

Within the human interactome, C.Prot entities tend to be both closer - in terms of shortest path distance - to themselves and to the non C.Prot proteins than on average in the whole human interactome, Figure 2A. This bias is increased within abundant C.Prot entities. It indicates that C.Prot is embedded in the interactome rather uniformly and does not constitute an isolated island, which certainly increases the robustness of the communications between C.Prot and the rest of the proteome through PPIs.

To better understand internal and external communication within and with C.Prot, we have introduced the central interactome and measured how it synchronizes certain biological processes (BPs) preferentially. Obviously, all the (true) PPIs are biologically relevant but our analysis aimed at identifying the main streams of communication. High-scoring exchanges (fluxes) between BPs provide a summary of the communications for which the interaction network is the most efficient. From Figure 3A we see that most exchanges in the central interactome are used to synchronize BPs with translation. BP communication between C.Prot and non C.Prot and outside the central interactome is no longer specialized and translation has a marginal role.

How well do we know these essential components found in C.Prot? Surprisingly, C.Prot contains 22 proteins lacking any information in PubMed (http://www.ncbi.nlm.nih.gov/), searching abstracts with gene symbols and their synonyms; 73 proteins appear in no more than 3 abstracts and 112 in no more than 6, see Suppl. Figure 6 and Suppl. Table 1.

## Conclusions

We have determined experimentally an approximation of the central human proteome that is suitable for analyzing its global properties. It is made of rather well conserved proteins which have gained additional expression flexibility through the acquisition of additional exons. These proteins are mainly involved in proteostasis, primary metabolism, cell cycle and death. They tend to be well connected with other proteins via PPIs. Random network simulations show that the central interactome was made very efficient, through evolution, to coordinate translation with other biological processes, or the latter ones via translation indirectly. More abundant proteins tend to be located at biological pathway central positions. Such central positions are generally devoid of drug targets, especially when they are part of the central proteome, which underline the fundamental role of these proteins whose activity should not be altered. Surprisingly, 10% of these common proteins are essentially uncharacterized. Our data can help other researchers to prioritize protein characterization or serve as background when analyzing focused datasets. They are made publicly available through the journal web site (Additional file 2) and a public repository: the complete lists of protein/peptide identifications in each cell line with spectra have been deposited in ProteomeCommons.org Tranche (hash= "JUrzEy1ShYDDUoKVrxHaoMrAu/CGbqv3xqOS/zuErFvlD8MOrVPRu5kOSlcxwK+/EYdA9WoLN5eMeprBzh9rPMIuYksAAAAAAAAPLQ=="); a more compact version of all the identifications found in each cell line in tabular format and without the spectra has also been deposited in Tranche (hash=" DrsqOg2DmUzUlJVLom+O6AHQTyJa2v+Ekhbw8az6OfF/JfF4hv51cyWqCKmaOHZZnOKJTUDl9ziTdCKpEzirhmjt9csAAAAAAAANKw==").

## Methods

### Proteomics

Total lysates of wild-type K562, HEK293, Namalwa, HaCat, HepG2, U937, HeLa cells (50 μg total protein) were reduced, alkylated, and separated by 1D SDS-PAGE. After visualization of the proteins by Coomassie Blue, entire gel lanes were sliced into 50 equal pieces and digested *in situ *with modified porcine trypsin [41]. The resultant peptide mixture was extracted from the gel slices and desalted with customized reversed-phase stage tips [42]. Each cell line was grown to complete confluence in appropriate media.

Approximately 10% of each tryptically-digested sample was analyzed as technical replicates by data-dependent nanocapillary reversed-phase LC-MSMS. Peptide separation was via customized 50 μm inner diameter columns packed with 3 μm diameter C18 Reprosil beads coupled to a hybrid LTQ-Orbitrap XL mass spectrometer (ThermoFisher Scientific, Waltham, MA). Data-dependent acquisition was performed for 100 min using one MS channel for every four MSMS channels and a dynamic exclusion for selected ions of 60 s.

### Protein identifications

Protein identification combined Mascot [25] and Phenyx [26], both with 4 ppm/0.3 Da parent/fragment mass tolerance, maximum 1 missed cleavage, carbamidomethyl cystein as fixed modification, and methionine oxidation variable, minimum peptide length 6 amino acids. Searches were performed against UniProtKB/Swiss-Prot (vers. 56.1) [29] human section, including all the isoforms. Results of the two engines were parsed separately and a minimum of 2 distinct peptides above a score threshold was required. Single peptide hits (SPHs) were also accepted but above a much higher score threshold and provided the protein sequence coverage was 2.5% or more, see hereafter. We discarded spectra assigned to different peptides by the two search engines during the merge of the results. The identified proteins passing this selection were grouped according to shared peptides, and groups with no specific peptides were discarded (1%), Suppl. Figure S7. Based on reverse database searches, we imposed a protein group FDR of 0.25%, see Suppl. Figure S8, which resulted in the following Mascot thresholds: 2 peptides with ion score 18 or more, export all additional peptides with score 10 or more, SPHs with ion score 50 at least; and Phenyx thresholds: 2 peptides with z-score 4.5 or more and P-value 0.001 or less, export all additional peptides with z-score above 3.5, SPHs z-score above 6.

We characterized the robustness of protein groups by determining the peptide false positive (FP) rate induced by the score thresholds we obtained constraining the protein groups FDR. We observed that this FP rate is < 0.1%. Since we discarded protein groups not having any specific peptide (Suppl. Figure 7), the probability that a group is nonetheless artifactual, but not detected as such because of a single FP specific peptide identification, is less than 0.1%.

We have identified more proteins with Phenyx than with Mascot (+4% on average) and with a better sequence coverage: 30% of the proteins have higher sequence coverage with Phenyx, whereas 15% have higher sequence coverage with Mascot. Phenyx scoring function used for this work is available as Additional File 3.

### Protein abundance estimation

It is well-known that spectral and peptide counts can provide reasonable estimates of protein abundance [43-45]. We decided to employ emPAI [45], which is a modified spectral count taking into account the instrument mass range, because it has been carefully validated by its authors and is well accepted. We used a mass range of 698 to 2370 Da that covered 99% of the detected peptides.

To separate the common presence from the sole abundance in the analyses below, we built a comparison dataset Top.CL representing the abundant proteins not commonly expressed. For each protein, we estimated its abundance in the 7 cell lines with emPAI and, to obtain a single number, we took the median. Then, considering the proteins that did not appear in more than 5 cell lines, Top.CL was defined as the proteins in the top 25% median emPAI (463 proteins). To distinguish between different levels of expression within C.Prot, we followed a similar procedure and defined Low.C.Prot (low 25%, 278 proteins) and Top.C.Prot (top 25%, 281 proteins).

### Protein interaction data and pathways

We have integrated five public databases containing human protein-protein interactions (PPIs) (MINT [46], IntAct [47], HPRD [48], BioGRID [49], protein complexes found in NCI-PID [36]) mapping all accession codes onto Swiss-Prot. From this integrated database, restricting to physical measurements of PPIs by tandem affinity purification (TAP), we extracted 70083 interactions between 9495 distinct human proteins and we built a network model of the human interactome; 859 proteins of C.Prot (76%) were found in this network.

Many measures have been proposed to characterize connectivity in networks [50]. We retained 5 well-established measures ranging from centrality of nodes to likely involvement in complexes and modules: Betweenness centrality [51], which measures the ratio of the number of shortest paths through a given node by the total number of shortest paths, i.e. how likely it is that a sequence of protein interactions - a pathway - goes through this node; Eigenvector centrality [52] measures centrality in dependence of neighbors own centrality, i.e. the centrality of a node increases when it is connect to nodes that are central themselves; Node degree is the number of neighbors of a node; Clustering coefficient [53] measures the ratio between the maximum number of edges theoretically possible in the sub-network spanned by a node and its immediate neighbors and the actual number of edges; *k*-core score measures the maximum number *k *for a node to be in a *k *-core [54]. A *k*-core is a sub-network with all nodes connected to at least *k *nodes of the sub-network.

To compare distributions of topological measures, such as the clustering coefficient above, for different sets of proteins, e.g. C.Prot versus C.Trans, we applied a goodness-of-fit test. Using classical χ^2 ^test yielded excessively small P-values since a lot of data points were available and very little differences became significant. To circumvent this problem, we re-sampled the data 1000 times, using 500 data points from each of the two sets to compare, and we computed the χ^2 ^statistic (10 bins). We thus obtained 1000 χ^2 ^statistics, took the median and obtained the P-value from the χ^2 ^distribution.

To investigate positions of proteins in biological pathways, we used the NCI-PID database [36], which comprises BioCarta (http://www.biocarta.com/) and Reactome [55] pathways in addition to NCI-PID unique pathways. We found 573 of our proteins (55%) in these pathways and we computed the relative position of each. The relative position was defined considering the shortest paths to the closest source and end nodes, see Suppl. Figure S5 for an example. If a protein appeared several times in a pathway, relative positions were averaged. We did not distinguish between isolated proteins and proteins in complexes, and occurrences of proteins in several pathways were averaged as well. Replacing the shortest path to the closest source/end nodes by the average over all the shortest paths to all the source/end nodes, which would be a reasonable alternative measure, we obtained nearly identical results (data not shown).

### GO fluxes

To measure fluxes between GO [31] biological processes (BPs) mediated by PPIs, we counted pairs of GO terms. Namely, given two proteins in interaction, P_1 _annotated with BPs A and B and P_2 _annotated with C, D, and E, we count 1 for the pairs AC, AD, AE, BC, BD, and BE. The counts are summed over all PPIs. To avoid "re-discovering" the GO structure, we removed all common ancestors of GO terms found at each protein.

To identify GO fluxes that were stronger than what would be expected as a result of the frequencies of GO terms and protein connectivity in the interactome, we generated 100 random interaction networks and GO annotations. We used 90 such random annotated networks to determine the means and standard deviations of random fluxes between BPs: mean_random_(BP_i_,BP_j_), sd_random_(BP_i_,BP_j_). Normalized scores were then defined as NScore(BP_i_,BP_j_) = (flux(BP_i_,BP_j_) - mean_random_(BP_i_,BP_j_))/sd_random_(BP_i_,BP_j_) and brought all terms to a common scale, independent of their frequencies. The last 10 random networks were used to learn the normalized scores null distribution, which is bell-shaped. Random networks were generated with topologies, GO term frequencies, and central proteome nodes matching the original data. We considered random networks, where node degrees and GO term frequencies were preserved individually but decoupled.

## Abbreviations

LC: liquid chromatography; MS: mass spectrometry; MSMS: tandem mass spectrometry; PPI: physical protein interaction; SPH: single peptide hit; GO: gene ontology; BP: biological process; emPAI: exponentially modified protein abundance index; C.Prot: central proteome; Top.CL: top 25% most abundant proteins not in the central proteome; Top.C.Prot: top 25% most abundant proteins in the central proteome; Low.C.Prot: low 25% abundant proteins in the central proteome; Spe.Trans: tissue-specific transcripts excluding testis; HPA: Human Protein Atlas

## Authors' contributions

TRB and JC performed the bioinformatics analysis. TRB, JC and GSF designed the project and wrote the manuscript. MP and KB realized the proteomics analysis of the samples. TB contributed to the project design and the interpretation of the results. IK did the cell culture work. FB implemented and adapted several in-house databases used for this project. All authors read and approved the final manuscript.

## Supplementary Material

Additional file 1 **Supplementary material**. Supplementary material contains several figures and tables that further support the results discussed in the paper. Click here for file

Additional file 2 **Central proteome list**. A table listing all the accession codes, IDs, description, and gene names of the 1124 proteins found in the central proteome. Click here for file

Additional file 3 **Linear trap Phenyx scoring function**. The scoring function used in this study that we developed for ThermoFisher linear traps. It can be added to any Phenyx installation. Click here for file
