# Supplementary material for: Initial characterization of the human central proteome
Source: BMC Syst Biol. 2011 Jan 26;5:17. doi: 10.1186/1752-0509-5-17 (PMC3039570; doi:10.1186/1752-0509-5-17)
Supplement: Additional file 1 — Supplementary material. Supplementary material contains several figures and tables that further support the results discussed in the paper. [file 1752-0509-5-17-S1.PDF]

Initial Characterization of the Human Central Proteome

Thomas R. Burkard, Melanie Planyavsky, Ines Kaupe, Tilmann Bürckstümmer, Keiryn L. Bennett, Giulio Superti-Furga, Jacques Colinge*

Supplementary Material

Tables and Figures complementing the main text

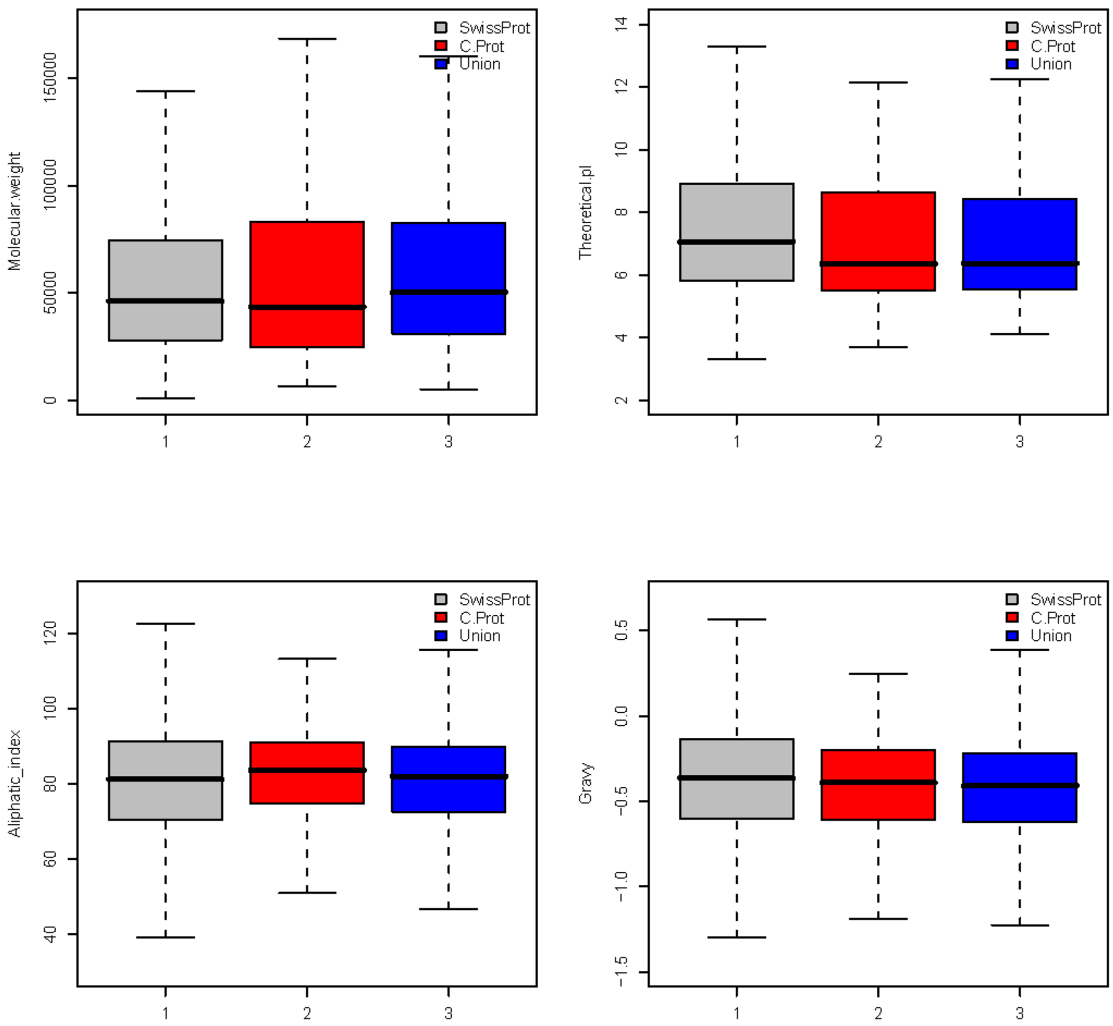

Figure S1: Experimental biases in the identified proteins. Taking UniProtKB/Swiss-Prot as a reference (gray), we see that the distributions of C.Prot (red) and the complete set of identified proteins (blue) do not deviate strongly. Only the molecular weights and the pI are slightly biased but these are normal consequences of the 1D gel protein separation and mass spectrometry analysis.

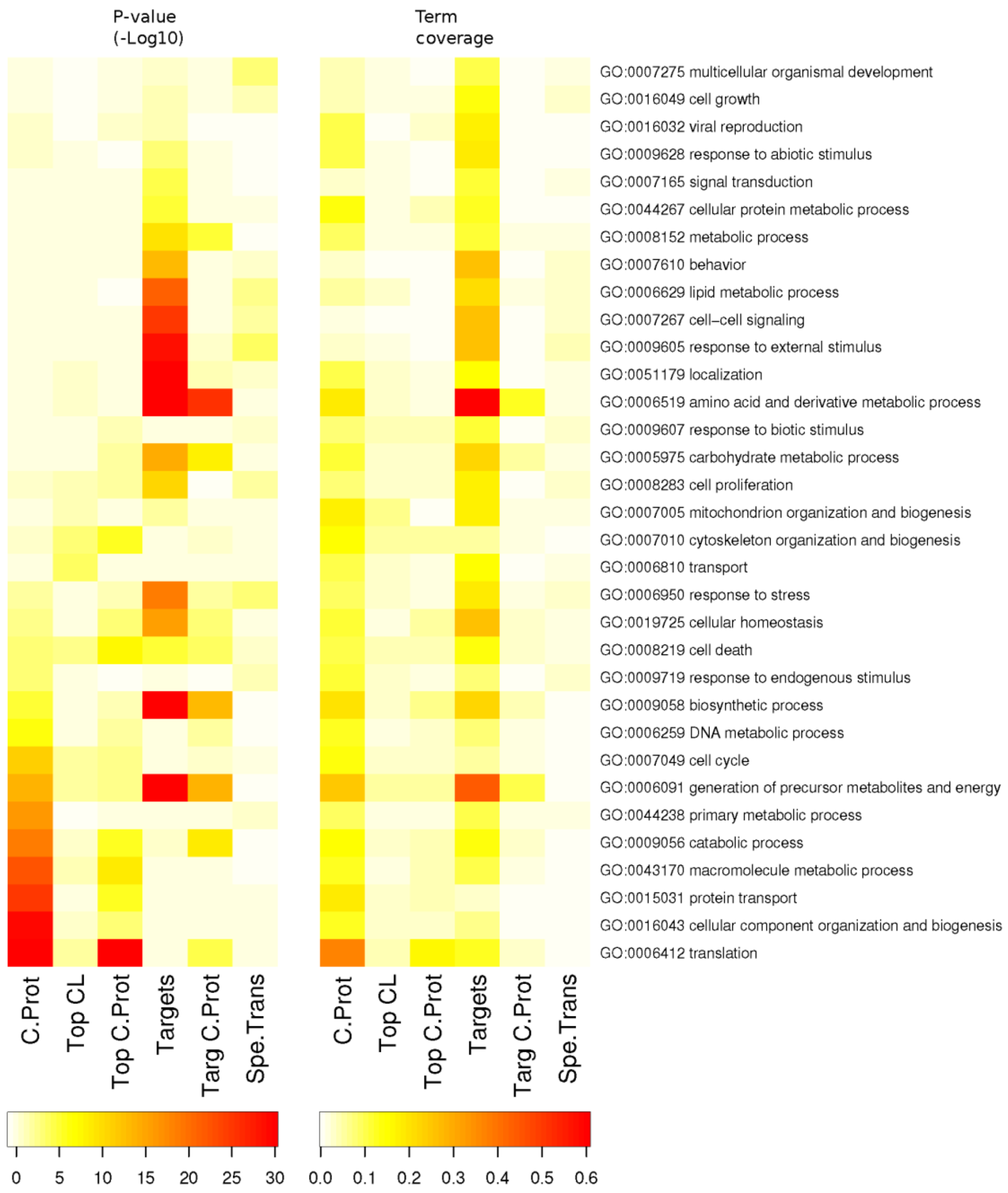

Figure S2: Biological process GO terms. We compare the different datasets discussed in the paper and report both the P-values of enrichment (left) according to a hypergemetric model and how well GO terms are covered (right). C.Prot stands for the central proteome, Top CL are the abundant proteins not expressed by more than 5 cell lines, Top C.Prot the abundant proteins of C.Prot, Targets are drug targets as listed by DrugBank, Targ. C.Prot are drug targets present in C.Prot, and Spe.Trans are tissue specific genes (orthogonal dataset to C.Prot).

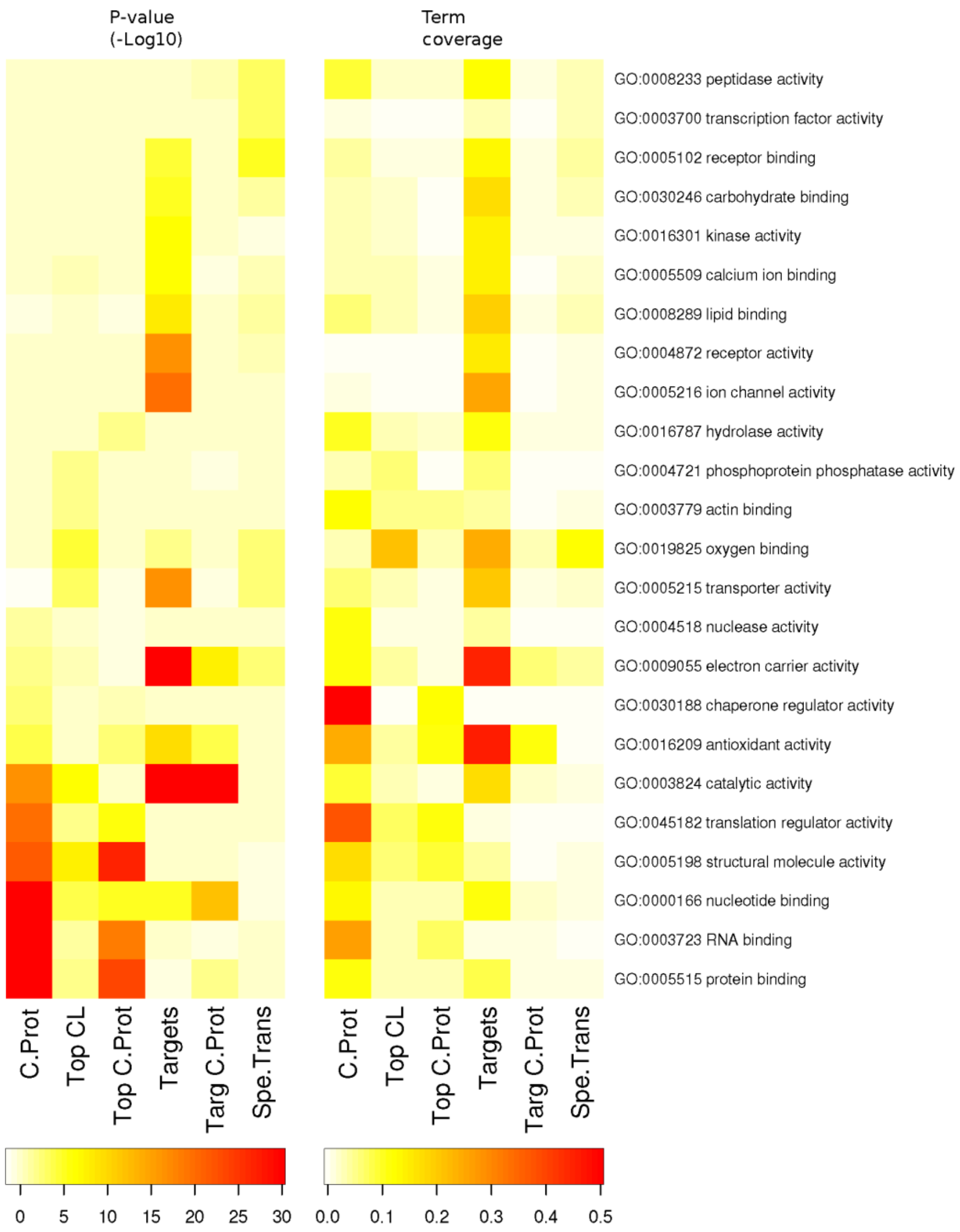

Figure S3: Molecular function GO terms.

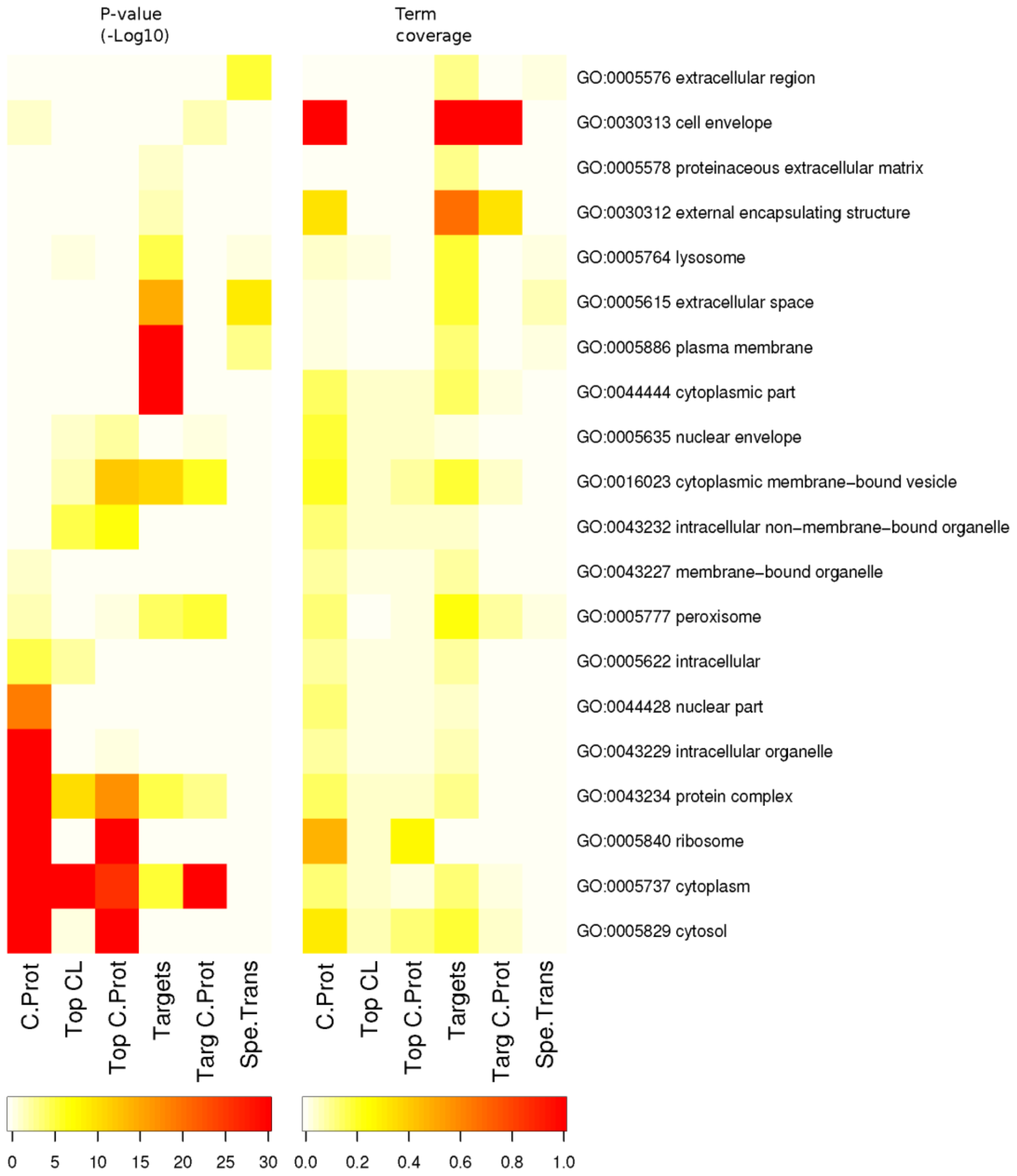

Figure S4: Cellular location GO terms.

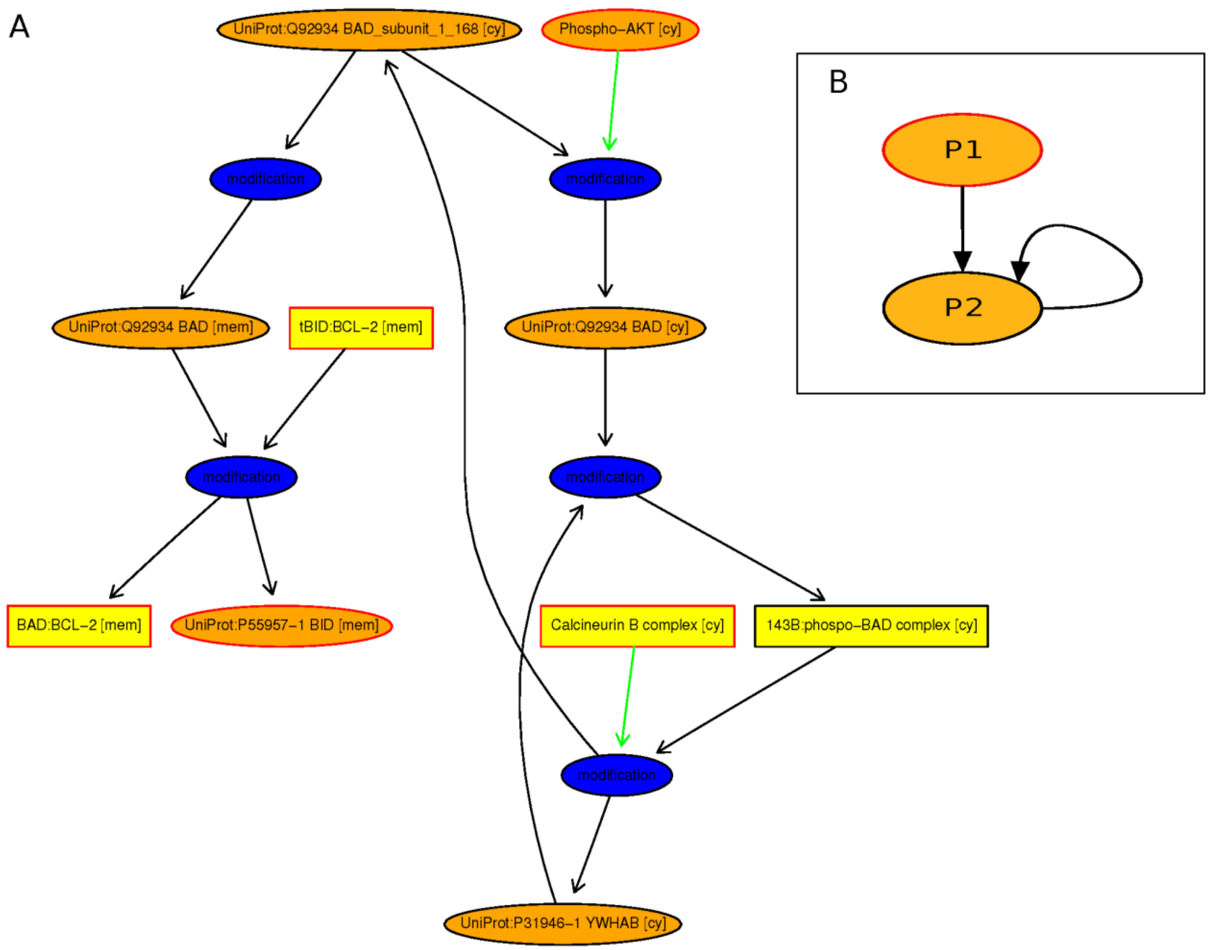

Figure S5: (A) Example of pathway (activation of BAD and translocation to mitochondria, Reactome) to illustrate relative position computations, orange=proteins, yellow=complexes, blue=reactions, green arrow=activation, red border=source/end node. YWHAB is at distance 2 from Calcineurin B complex, the closest source node, and at distance 8 from BAD:BCL-2 and BID, the 2 closest end nodes. Hence, its relative position is (distance to source)/(distances to source + end) = 2/(2+8) = 0.2. Now, if instead of the shortest paths to the closest source and end nodes we use the average we have distances to sources 2 (Calcineurin B complex), tBID:BCL-2 complex is not possible (directed network), and 6 (AKT), and to ends 8 (BAD:BCL-2 complex), 8 (BID). Therefore, the relative position would then be (average distance to sources)/(average distance to sources + average distance to ends) = 4/8 = 0.5. (B) In case of no end node accessible, alternatively no source node, nodes such as P2 are at distance 0 to an end, and thus P1 would have relative position 0 and P2 relative position 1.

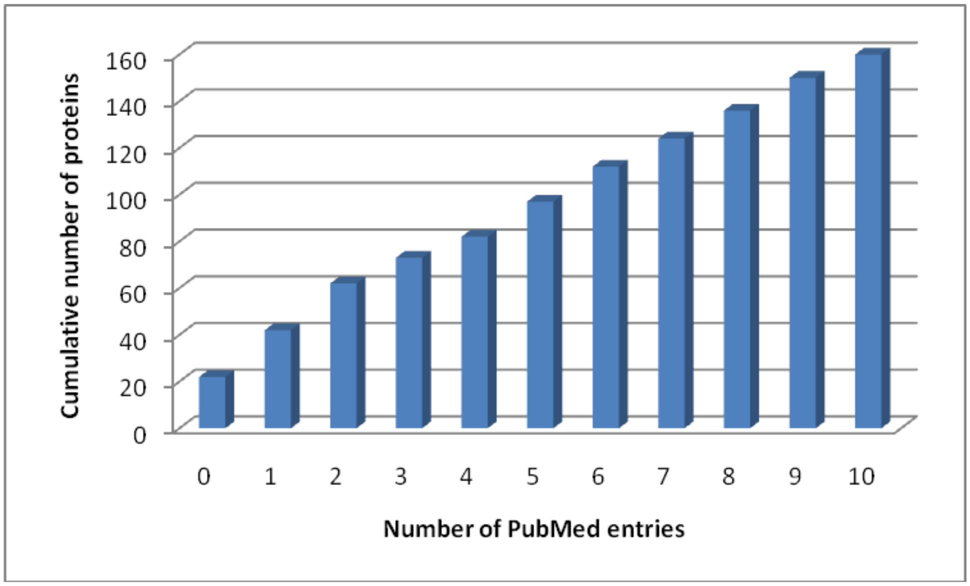

Figure S6: Cumulative number of proteins in C.Prot with no more than 10 abstracts found in PubMed, searching with the official gene symbols and their synonyms.

Table S1: UniProt primary accession codes of the protein in C.Prot having no more than 5 abstracts in PubMed.

AC	Number of Abstracts	AC	Number of Abstracts	AC	Number of Abstracts	AC	Number of Abstracts
O60361	0	Q9Y3B3	1	Q92552	2	Q9UIA9	4
Q96AG4	0	Q9BVK6	1	O75438	2	P82932	4
Q9GZN8	0	Q5T8P6	1	Q96GC5	2	Q6UB35	4
Q9NUQ9	0	Q9Y3D9	1	Q3MHD2	2	Q9UPU5	4
Q9BQ61	0	Q9H773	1	O75937	2	Q9P2R3	4
Q8N766	0	Q9NVI7	1	Q9H3K6	2	O43760	4
Q6P587	0	Q9Y399	1	Q9Y520	2	P56385	4
Q9BPX5	0	Q9NUU7	1	Q9P0M9	2	Q9H3K2	5
Q5T6F2	0	O75947	1	P09661	2	O60524	5
Q9Y3B4	0	Q9H2W6	1	O75153	2	O43837	5
Q6GMV3	0	Q9BWJ5	1	Q8N983	2	Q9Y5M8	5

Q9Y2R0	0	Q9UMY4	1	P29084	2	Q9Y320	5
Q9BV86	0	O96000	1	O95782	3	Q9NUQ8	5
O75390	0	Q4VC31	1	Q9Y262	3	Q9UI30	5
Q9BRJ6	0	Q7Z4H3	1	O43809	3	Q8TBX8	5
Q7Z4V5	0	P57088	1	Q9Y3I0	3	Q9Y3B7	5
Q9BVC6	0	Q15006	1	Q7Z478	3	Q7Z2Z2	5
Q96A33	0	Q9NX55	2	Q7L1Q6	3	Q96K76	5
Q96C36	0	Q7L2E3	2	Q5T1M5	3	Q9BQ67	5
Q9GZP4	0	Q02543	2	P24539	3	P36542	5
Q13151	0	Q9UHV9	2	Q8WU90	3	Q9HC38	5
Q86Y56	0	Q8N6L1	2	Q9NRX1	3	P31937	5
Q14166	1	Q9Y6E2	2	Q96IX5	3		
Q9NPA0	1	P30049	2	Q9UI26	4		
O43169	1	O75964	2	Q15070	4		

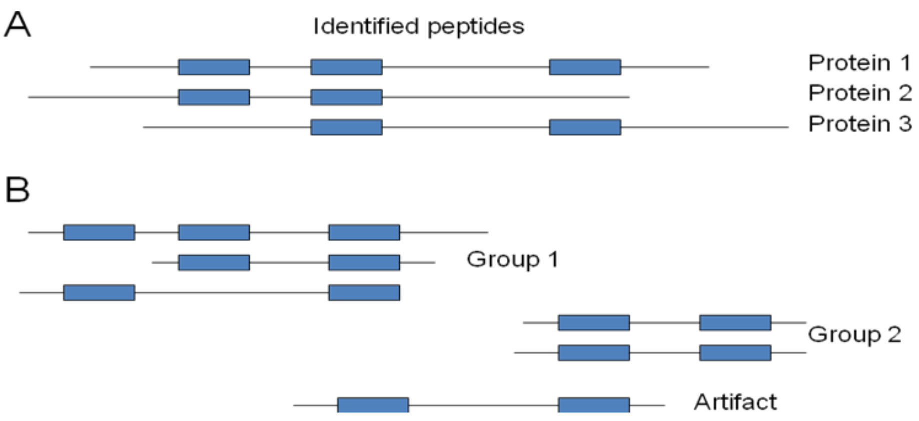

Figure S7: Possible structures of protein groups. (A) Standard configuration with protein 1, the group reporter, having maximum experimental evidence. Presence of proteins 2 and 3 cannot be excluded. It is even possible to imagine that proteins 2 and 3 only are present and protein 1 not, although it is less likely than the commonly accepted scenario of protein 1 presence. (B) How two groups can overlap to create an artifactual third group (analogue to presence of proteins 2 and 3 but not 1 in panel A).

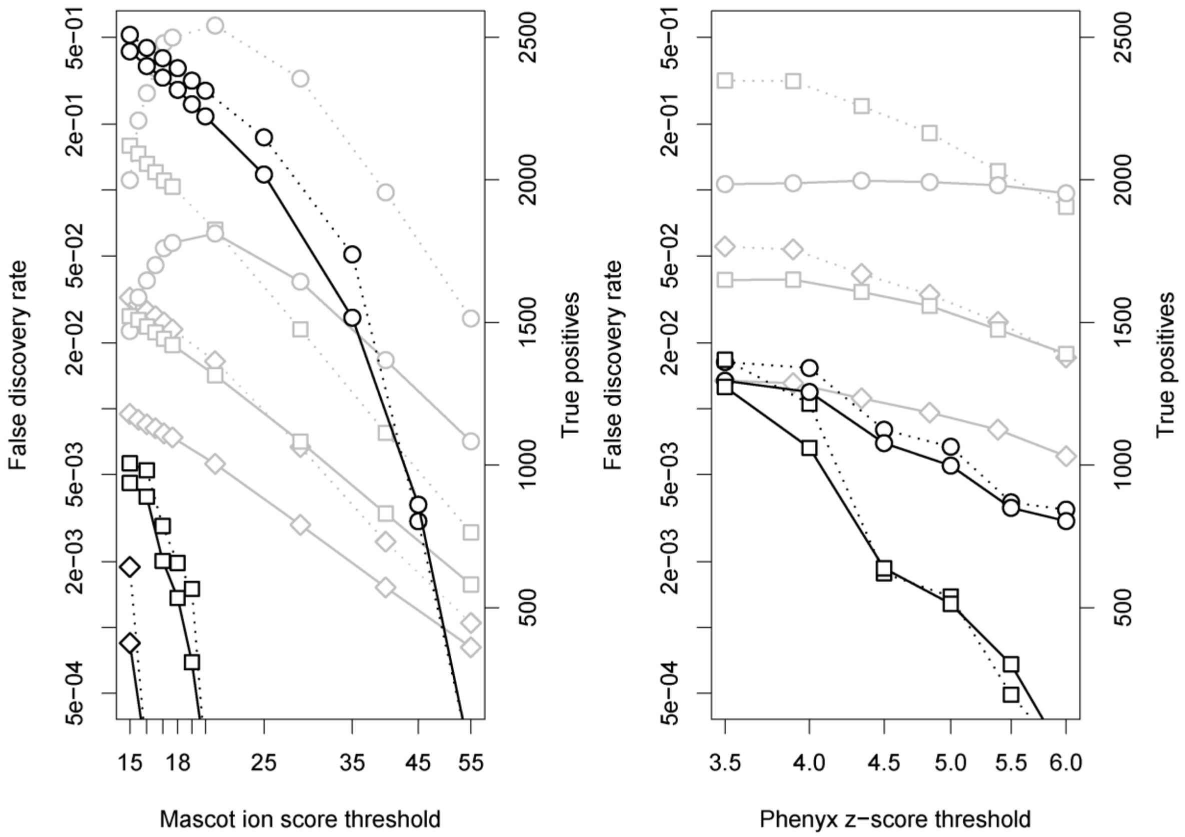

Figure S8: Illustration of the protein FDR dependency (solid black) with respect to the minimum peptide score thresholds and the number of distinct peptides required. Circles represent single peptide hits, squares hits with 2 distinct peptides at least, and diamonds hits with 3 peptides at least. Diamonds are not visible in the left-panel as no false positive protein group was detected. Solid gray curves represent the number of protein groups identified (True positives, right-hand y-scale). We make several observations: more protein groups are found requiring 2 peptides for FDR 0.25%; note that Phenyx is much less false positive prone in single peptide hits. Dashed curves report corresponding results on protein, as opposed to protein group, identifications.
